# Comment on ‘The Values and Preferences of People Living With Motor Neurone Disease (MND): A Systematic Review and Meta‐Analysis’

**DOI:** 10.1002/gin2.70088

**Published:** 2026-09-22

**Authors:** Rachna Rohtagi, Parag Jayant Ratnakar, Deepti M. Sati, Hariharan Srinivasan

**Affiliations:** ^1^ Department of Anatomy School of Medical Sciences and Research, Sharda University Greater Noida India; ^2^ Dr. D. Y. Patil Medical College Hospital and Research Centre, Dr. D. Y. Patil Vidyapeeth (Deemed‐to‐be‐University) Pune Maharashtra India; ^3^ Faculty of Pharmaceutical Sciences Graphic Era Hill University Dehradun India; ^4^ Centre for Promotion of Research Graphic Era Deemed University Dehradun India; ^5^ Saveetha Medical College and Hospital Saveetha Institute of Medical and Technical Sciences, Saveetha University Chennai India

Dear Editor,

Barker et al. systematic review with meta‐analysis provides a valuable synthesis of outcomes that matter to people living with motor neurone disease (MND) [1]. Before these findings are used to prioritise outcomes for the Australian MND Guideline, however, two linked issues merit clarification: whether all included measures capture values and preferences, and which synthesis model underlies the stage‐specific utility estimates.

Under the GRADE framework, values and preferences refer to the relative importance people place on health outcomes [2, 3]. Several conclusions in the review, however, are derived from ALS‐specific quality‐of‐life symptom scores reported by Raheja et al. [4]. In that source study, each symptom was scored from 0 (no problem) to 10 (a tremendous problem), quantifying perceived symptom burden over time rather than choices, trade‐offs, rankings or utility valuations. Interpreting changes in eating, mucus, bowel and bladder, sleep or other problem scores as preferences for interventions risks conflating burden with priority. A highly troublesome symptom may not be prioritised when survival, communication, autonomy, adverse effects and treatment burden compete, and the converse is also possible.

The King's‐stage analysis also requires reconciliation. The Results text reports broad stage‐specific intervals, including a stage 4 estimate of 0.31 (95% CI −0.07 to 0.69), whereas Table 1 and Figure 2 report 0.40 (95% CI 0.36 to 0.44). The methods specify fixed‐effect models when three or fewer studies contribute, but the Figure 2 caption describes a random‐effects model; only two studies contributed. These differences are consequential: one estimate is highly uncertain and includes health states valued below death, while the other appears precise. The statement that sensitivity models produced no important difference, therefore, needs explanation and explicit identification of the primary analysis.

A constructive refinement would stratify the evidence by elicitation construct: preference‐based utility and direct‐choice or ranking methods; symptom‐burden or problematicness scores; and generic or disease‐specific health‐status measures. Guideline judgments about outcome importance should rely principally on construct‐valid preference evidence, while burden measures can provide complementary context. For King's staging, fixed‐ and random‐effects estimates should be shown side by side, the primary model and variance assumptions identified and model‐dependent uncertainty reflected in the GRADE assessment. These clarifications would strengthen the review's contribution and make its evidence more interpretable for patient‐centred outcome selection.

## Author Contributions

**Rachna Rohtagi:** conceptualisation, investigation, funding acquisition, writing – original draft. **Parag Jayant Ratnakar:** methodology, validation, writing – review and editing, visualisation. **Deepti M. Sati:** software, formal analysis, project administration. **Hariharan Srinivasan:** data curation, supervision, resources.

## Funding

The authors have nothing to report.

## Conflicts of Interest

The authors declare no conflicts of interest.

## Data Availability

The data that support the findings of this study are available on request from the corresponding author. The data are not publicly available due to privacy or ethical restrictions.

## References

[1] T. H. Barker , L. N. Bulto , G. Holland , et al., “The Values and Preferences of People Living With Motor Neurone Disease (MND): A Systematic Review and Meta‐Analysis,” Clinical and Public Health Guidelines 3 (2026): e70080, 10.1002/gin2.70080. PMC13563401 42740868

[2] Y. Zhang , P. A. Coello , J. Brożek , et al., “Using Patient Values and Preferences to Inform the Importance of Health Outcomes in Practice Guideline Development Following the GRADE Approach,” Health and Quality of Life Outcomes 15 (2017): 52, 10.1186/s12955-017-0621-0. PMC5412036 28460638

[3] Y. Zhang , P. Alonso‐Coello , G. H. Guyatt , et al., “GRADE Guidelines: 19. Assessing the Certainty of Evidence in the Importance of Outcomes or Values and Preferences: Risk of Bias and Indirectness,” Journal of Clinical Epidemiology 111 (2019): 94–104, 10.1016/j.jclinepi.2018.01.013. 29452223

[4] D. Raheja , H. E. Stephens , E. Lehman , S. Walsh , C. Yang , and Z. Simmons , “Patient‐Reported Problematic Symptoms in an ALS Treatment Trial,” Amyotrophic Lateral Sclerosis and Frontotemporal Degeneration 17, no. 3/4 (2016): 198–205, 10.3109/21678421.2015.1131831. PMC4979603 26824413

